# Cord Blood as a Source of Natural Killer Cells

**DOI:** 10.3389/fmed.2015.00093

**Published:** 2016-01-05

**Authors:** Rohtesh S. Mehta, Elizabeth J. Shpall, Katayoun Rezvani

**Affiliations:** ^1^Division of Hematology, Oncology and Transplantation, University of Minnesota Medical Center, Minneapolis, MN, USA; ^2^Department of Stem Cell Transplantation, Division of Cancer Medicine, The University of Texas MD Anderson Cancer Center, Houston, TX, USA

**Keywords:** cord blood, natural killer cells, adoptive immunotherapy, KIR mismatch, transplantation, CBT

## Abstract

Cord blood (CB) offers several unique advantages as a graft source for hematopoietic stem cell transplantation (HSCT). The risk of relapse and graft vs. host disease after cord blood transplantation (CBT) is lower than what is typically observed after other graft sources with a similar degree of human leukocyte antigen mismatch. Natural killer (NK) cells have a well-defined role in both innate and adaptive immunity and as the first lymphocytes to reconstitute after HSCT and CBT, and they play a significant role in protection against early relapse. In this article, we highlight the uses of CB NK cells in transplantation and adoptive immunotherapy. First, we will describe differences in the phenotype and functional characteristics of NK cells in CB as compared with peripheral blood. Then, we will review some of the obstacles we face in using resting CB NK cells for adoptive immunotherapy, and discuss methods to overcome them. We will review the current literature on killer-cell immunoglobulin-like receptors ligand mismatch and outcomes after CBT. Finally, we will touch on current strategies for the use of CB NK cells in cellular immunotherapy.

## Introduction

Cord blood (CB) is a rich source of hematopoietic stem and progenitor cells and is being increasingly used as a graft source for hematopoietic stem cell transplantation (HSCT) (1). Despite the naive nature of CB T-lymphocytes (2, 3), the risk of relapse is lower after cord blood transplantation (CBT) compared with other donor sources (4–7). As natural killer (NK) cells are the first lymphocytes to reconstitute after HSCT and CBT (8–14) and their role in both innate and adaptive immunity is well characterized (15–17), it is likely that they play a crucial role in protection against early disease relapse. This article focuses on the uses of CB as a source of NK cells in CBT as well as in adoptive immunotherapy.

## Natural Killer Cell Phenotype

Natural killer cells are immunophenotypically characterized as CD3^−^CD56^+^ lymphocytes and are broadly classified into the less differentiated CD56^bright^ or the mature CD56^dim^ populations (16, 18). CD56^bright^ cells have low or absent expression of CD16 and killer-cell immunoglobulin-like receptors (KIR), whereas the CD56^dim^ subset expresses both CD16 and KIR (18, 19). Resting peripheral blood (PB) CD56^bright^ cells are poorly cytotoxic but are potent secretors of immunomodulatory cytokines and have tremendous proliferative capacity in response to IL-2. Conversely, PB CD56^dim^ cells are highly cytotoxic but have a poor capacity to proliferate in response to cytokines (16, 18–26).

## How Do Cord Blood NK Cells Differ from Peripheral Blood NK Cells?

Cord blood offers unique advantages, many of which are directly applicable to NK cell-directed alloreactivity. The ease of collection of CB and cryopreservation makes them readily available as an off-the-shelf source for NK cell immunotherapy (27, 28). Besides, the presence of almost a log fewer T-cells in CB compared to other graft sources (29–33), most of which are naive (34–36), minimizes the risk of graft vs. host disease (GVHD) (7, 29, 33, 37–40). More importantly, NK cell reconstitute more rapidly after CBT than PB HSCT (41, 42). One study showed that the absolute numbers of CD56 CD16 NK cells were significantly higher for up to 2 years after double unit CBT compared with filgrastim-mobilized unrelated donor PB HSCT (11). Moreover, CB contains unique cell populations, which represent NK-cell progenitors and are either absent or present in minute numbers in PB (43–49). These cells have a potential to differentiate into NK cells after *ex vivo* stimulation with cytokines, including IL-2 (48), IL-15, and/or FLT-3 ligand (43, 47, 48). Data also suggest that CB CD56^bright^ NK cells (but not CB T-cells) produce significantly more IFN-γ after stimulation with IL-12 and IL-18 compared with PB NK cells (36). This may in turn compensate for the hypofunctionality of naive CB T-cells – thus also contributing to a lower risk of GVHD while maintaining the crucial graft vs. leukemia effect. After stimulation with IL-12 and IL-18, the expression of CD69 (an activation marker) is increased appreciably on CB NK, but not PB NK cells (36). Moreover, the expression of CXCR4, a bone marrow (BM) homing receptor, is significantly higher in CB CD56^bright^ and CD56^dim^ NK cells compared with their PB counterparts (50), suggesting that CB NK cells may have better BM homing potential.

## Limitations of Cord Blood as a Source of NK Cells

There are also noteworthy limitations to the use of unmanipulated CB as a source of NK cells for immunotherapy. The foremost impediment relates to the finite number of NK cells available in a single CB unit. Although the frequencies of NK cells in PB and CB are similar (50–53), the small volume of blood in a CB unit makes it challenging to obtain adequate numbers needed for clinical use. A second crucial obstacle is the functional immaturity of resting CB NK cells. In contrast to PB, CB NK cells express very few inhibitory KIRs, have a higher expression of the inhibitory receptor NKG2A and almost completely lack CD57 expression, an activation marker associated with terminal differentiation of NK cells (49, 50, 54, 55). Moreover, the expression of other activation receptors, such as NKp46, NKG2C, and DNAM-1, are lower in CB NK cells (50). As a result, resting CB CD56^dim^ NK cells have poor *in vitro* cytotoxicity compared with PB NK cells.

To overcome these limitations, a number of groups have developed *ex vivo* expansion techniques that can increase NK cell numbers by about 1800- to 2400-fold from either fresh or cryopreserved CB units (56). NK cells can also be successfully differentiated from CB CD34^+^ cells (57–60) using a cocktail of cytokines and membrane-bound IL-15 (60). Most expansion techniques use IL-2 either alone (61, 62) or in combination with IL-15 (63), or IL-7 (64), or stem cell factor and FLT3-ligand, (64) or a supporting layer of mesenchymal stromal cells (65), or artificial antigen-presenting cell, such as K562 cells expressing membrane-bound IL-21 (56). Expansion techniques not only augment CB NK cell numbers but also result in the acquisition of functional competence and similar activity to *ex vivo* activated PB NK cells (56).

## NK Cell Alloreactivity

The alloreactivity of NK cells is guided by a fine balance between their activating and inhibitory receptors, and interactions with their cognate ligands. The inhibitory KIRs recognize classical MHC-I molecules (HLA- A, -B, and -C) C-type lectin family of receptors (CD94 and NKG2s – NKG2A, -B, -C, -D, -E, and -F) recognize non-classical MHC-I molecules (HLA-E and stress-induced MHC-I related chains – MICA and MICB), while the ligands for natural cytotoxicity receptors (NKp46, NKp30, NKp44, NKp80, and others) and activating KIRs are largely unknown [reviewed in Ref. (66–69)]. The recognition of “self” MHC-I molecules on normal cells by inhibitory NK receptors protects them from NK cell-mediated lysis (70, 71). However, malignant or infected cells often shed or down-regulate their MHC-I molecules as an immune escape mechanism (72, 73), which revokes NK cell inhibition and triggers the activating receptors to cause cell lysis (74, 75). This principle could be exploited to our advantage in HSCT because the human leukocyte antigen (HLA) system (chromosome 6) and KIR genes (chromosome 19q13.4) are located on different chromosomes and segregate independently (76, 77). This creates a possible scenario of donor–recipient HLA-match appropriate for HSCT, yet retaining mismatch in KIRs and their ligands, yielding alloreactive NK cells against the recipient tumor cells.

## Different Models Used to Define NK Cell Alloreactivity

Before reviewing the clinical applicability of this concept, it is important to understand various methods used to assess NK cell alloreactivity. These include (a) testing donor NK KIR expression (genotype or phenotype) and the corresponding HLA [KIR-ligands (KIR-Ls)] in recipient, known as the *receptor–ligand model*, (b) determining HLA class I typing (KIR-Ls) in both donor and recipient, known as the *ligand–ligand model*, or (c) performing functional analysis of NK cell alloreactivity, where donor NK cells are tested for their ability to lyse a cell line or the recipient’s leukemia cells, known as the *cytotoxicity model* (78–81). Likewise, the KIR repertoire in an individual can be determined based on either (a) KIR genotype (DNA-based methods to assess KIR genes, or real-time quantitative PCR to assess mRNA expression) or (b) KIR phenotype (using flow cytometry for surface protein expression), although most currently available monoclonal antibodies used for flow cytometry cannot differentiate activating from inhibitory KIRs. To make matters even more complicated, about one-quarter of individuals have discrepancies in the genotype and phenotype of four inhibitory KIRs (KIR2DL1, KIR2DL2, KIR2DL3, and KIR3DL1) (82). For instance, the gene for KIR2DL1 is present in 93–97% of individuals, yet the corresponding receptor is absent in 7%, one or both allelic forms of KIR2DL2/KIR2DL3 exist in all individuals, and the gene for KIR3DL1 is present in about 90–92% of individuals, but about 15% of them do not express the corresponding receptor (80, 82).

Various studies evaluated transplant outcomes, using different models to predict NK cell alloreactivity. The earliest clinical evidence for the importance of NK alloreactivity in reducing the risk of relapse was provided by Ruggeri et al. (78, 83) in T-deplete haploidentical HSCT, using the KIR-L mismatch model to predict alloreactivity. Subsequent studies in the settings of haploidentical, matched or mismatched unrelated or unrelated donor HSCT produced surprisingly variable results, ranging from no advantage (84–88) to mixed response based on the type of KIR-L incompatibility model used for the analysis (81), to improvements in one or all aspects of relapse risk, disease free survival (DFS), or overall survival (OS) (79, 80, 89–93). However, caution is warranted in interpreting the results due to remarkable heterogeneity of these studies.

## Alloreactive NK Cells in Cord Blood Transplantation

Given the unique advantages offered by CB, four relatively large studies assessed the role of KIR-L mismatch in the CBT setting (Table 1) (94–97). All but one (97) failed to demonstrate a beneficial effect of KIR-L mismatch on disease outcomes after CBT. One study (94) rather reported that KIR-L mismatch was associated with higher risk of acute GVHD, and worse treatment-related mortality (TRM) and OS after reduced intensity conditioning CBT (RIC-CBT). All of these studies used the KIR-L mismatch model proposed by Ruggeri et al. (78).

**Table 1:** **Studies assessing the role of KIR-ligand mismatch in cord blood transplantation**.

			Brunstein et al. (94)				Tanaka et al. (96)					
	Willemze et al. (97)		Myeloablative conditioning		Reduced intensity conditioning		ALL		AML		Garfall et al. (95)	
	KIR-L matched	KIR-L mismatched	KIR-L mismatched	KIR-L matched	KIR-L mismatched	KIR-L matched	KIR-L mismatched	KIR-L matched	KIR-L mismatched	KIR-L matched	KIR-L matched	KIR-L mismatched
*N*	149	69	41	114	33	69	227	59	288	69	45	35
Age, median (range)	12.8 (0.6–69)	15 (0.5–64)	15 (0.6–53)	15.9 (1.0–59)	48 (22–69)	52 (6–68)	27	33	47	50	49 (24–67)	47 (21–65)
RI conditioning	25 (18)	10 (16)	0%	0%	100%	100%	47 (21)	11 (19)	101 (35)	28 (41)	34 (75.56)	25 (71.43)
CMV positive [*n* (%)]	?	?	14 (34)	73 (64)**	14 (42)	44 (64)**					43 (95.56)	29 (82.86)
ATG/ALG	106 (79)	52 (84)	17 (41)	43 (38)	8 (24)	22 (32)	0%	0%	0%	0%	100%	100%
Median infused CD3^+^ dose [×10^7^ (range)]	?	?	1.2 (0.1–2.6)	1.3 (0.2–3.2)	1.1 (0.1–2.7)	1.4 (0.2–3.1)	–	–	–	–	–	–
Single-CBT (%)	100	100	66	56	100	100	100	100	100	100	0	0
Graft rejection											1/45	5/35**
Grade II-IV GVHD [% (95% CI)]	30 ± 3%	28 ± 5%	46 (30–64)	46 (36–56)	79 (59–99)	57 (44–70)**					22%	17%
Grade III-IV GVHD [% (95% CI)]			28 (8–48)	17 (6–28)	42 (27–59)	13 (5–21)**	HR 1.06, *p*-value 0.83		HR 0.84, *p*-value 0.51			
Chronic GVHD at 1–2 years	(No difference)	(No difference)	10 (1–19)	21 (13–29)	12 (1–23)	14 (6–22)					19%	24%
% NRM/TRM	31 ± 4%	25 ± 5%	27 (14–40)	18 (11–25)	27 (12–42)	12 (5–19)**						
	RR 0.6 (0.31–1.16), *p*-value 0.13						HR 0.71 (95% CI 0.37–1.39), *p*-value 0.32		HR 0.95 (95% CI 0.52–1.72), *p*-value 0.86			
% Relapse at 2–3 years	37 ± 4%	20 ± 5%**	18 (6–30)	28 (19–37)	39 (21–57)	47 (34–60)					40%	44%
	RR 0.53 (95% CI 0.28–0.99), *p*-value 0.05**						HR 0.95 (95% CI 0.43–2.10), *p*-value 0.91		HR 0.59 (95% CI 0.31–1.14), *p*-value 0.12			
% Disease free survival at 2–3 years	40 ± 4%	55 ± 7%**									29%	24%
	RR 2.05 (95% CI 1.31–3.2), *p*-value 0.0016**						HR 0.79 (95% CI 0.49–1.29), *p*-value 0.352		HR 1.02 (95% CI 0.65–1.59), *p*-value 0.945			
% Overall survival at 2 years	31 ± 4%	57 ± 7%**	50 (32–68)	57 (47–67)	32 (15–59)	52 (47–67)**					40%	34%
	RR 2.00 (95% CI 1.24–3.22), *p*-value 0.004**						HR 0.87 (95% CI 0.53–1.40), *p*-value 0.557		HR 0.93 (95% CI 0.58–1.49), *p*-value 0.752			
Median (range) follow-up	13 months	15 months	2.2 (1.0–6.8) years	2.1 (0.9–7.8) years	2.0 (1.0–3.5) years	1.8 (0.9–5.3) years					34 months	34 months

**Significant p-value.

*ALL, acute lymphoblastic leukemia; AML, acute myeloid leukemia; ALG, anti-lymphocyte globulin; ATG, anti-thymocyte globulin; CBT, cord blood transplantation; CI, confidence interval; CMV, cytomegalovirus; GVHD, graft vs. host disease; HR hazards ratio; KIR, killer-cell immunoglobulin-like receptors; NRM, non-relapse mortality; RI, Reduced intensity; TRM, treatment-related mortality; ?, unknown*.

The first and the only CBT study to demonstrate any favorable effect of KIR-L mismatch was reported by Willemze et al. (97). The authors also included HLA A3/A11 (ligand for KIR3DL2) mismatches in their study, the role of which is quite controversial (98), because the interaction between KIR3DL2 and HLA-A3/A11 occurs in the presence of specific viral peptides (99) and the evidence of their *in vivo* interaction is scare (100). The study included 149 KIR-L matched and 69 KIR-L mismatched donor–recipient pairs, with median recipient ages of 12.8 and 15 years, respectively. Only patients with AML or ALL were included, all of whom received single unit CBT. The majority received myeloablative conditioning (MAC) and ATG were used in 79 and 84% of patients, respectively. There were no differences in the incidence of acute or chronic GVHD or non-relapse mortality (NRM) between the KIR-L matched and mismatched groups. However, the probabilities of 2-year relapse (37 vs. 20%, *p* = 0.03), 2-year DFS (40 vs. 55%, *p* = 0.005), and 2-year OS (31 vs. 57%, *p* = 0.02) were all significantly improved in the KIR-L mismatched group. In subgroup analysis, these differences were significant for patients with AML only. Furthermore, deaths due to opportunistic infections were more frequent in the KIR-L matched group (38 vs. 7%, *p* = 0.03). The median follow-up period (13 and 15 months) was relatively short, but the study findings remained unchanged in a later update with a median follow-up of 34 months (101).

In contrast, the Minnesota group (94) reported a detrimental impact of KIR-L mismatch in recipients of RIC CBT. The study included 183 KIR-L matched and 74 KIR-L mismatched donor–recipient pairs with a variety of hematological malignancies, including acute or chronic leukemias and lymphomas. Patients received either single or double unit CBT, the definition of KIR-L mismatch in the recipients of double CBT was arbitrarily defined based on the KIR-L of the dominant engrafting unit. Due to significant differences in the MAC and RIC groups, the results of these groups were reported separately. The MAC arm constituted primarily pediatric patients with median ages of 15 and 15.9 years in KIR-L matched and mismatched groups, respectively. The majority received single CBT (66 and 56%) and ATG were used sparingly (41 and 38%), respectively. As expected, patients who received RIC regimens were older with median ages of 48 and 52 years, respectively, and all underwent double unit CBT. Again, ATG was used in only a minority of patients (24 and 32%, respectively). The follow-up duration (medians 1.8–2.2 years in all groups) was longer than in the Willemze study (97). This study did not find an impact of KIR-L mismatch on any of the clinical outcomes after MAC, including acute or chronic GVHD, TRM, relapse risk, or OS. Conversely, in the RIC group, KIR-L mismatch was associated with significantly higher rates of grades II–IV (79 vs. 57%, *p* = 0.01) and grades III–IV (42 vs. 13%, *p* ≤ 0.01) acute GVHD, worse TRM (27 vs. 12%, *p* = 0.03), and OS (32 vs. 52%, *p* = 0.03). In multivariate analysis, KIR-L mismatch was the only significant predictor of higher rates of grades III–IV acute GVHD (RR 1.8, 95% CI 1.1–2.9; *p* = 0.02) and risk of death (RR 1.8; 95% CI 1.0–3.1, *p* = 0.05). Restricting their analysis to only AML patients, the authors again found higher incidence of grades III–IV acute GVHD in patients with KIR-L mismatch. Analysis performed with or without the inclusion of HLA-A3/A11 mismatches did not affect clinical outcomes.

Similar findings were reported in the double CBT setting by the Boston group (95) without the inclusion of HLA-A3/A11 mismatches. They included patients with diverse hematological malignancies, three-quarters of whom received RIC regimens with fludarabine, melphalan, and ATG. The study found no impact of KIR-L mismatch on the incidence of acute or chronic GVHD, relapse, DFS, or OS. However, the KIR-L mismatched group experienced more graft rejections (5/35) compared with KIR-L matched group (1/45, *p* = 0.08). Interestingly, 4/5 patients with graft rejection in the KIR-L mismatched group had cord vs. cord KIR-L mismatches and 3/5 had host vs. cord KIR-L mismatches. Later, a Japanese study (96) also found no impact of KIR-L (including HLA-A3/A11) mismatch in single unit CBT recipients with either AML or ALL. Of note, more than 80% of patients received TBI-based MAC regimens, but ATG was not used.

As noted, these studies are fairly heterogeneous in terms of their study population, underlying disease, use of single vs. double CB grafts, conditioning regimens, use of *in vivo* T-cell depletion, GVHD prophylaxis, inclusion of HLA-A3/A11 mismatch in the analysis, to name a few. The use of lymphodepleting chemotherapy regimens, such as high-dose cyclophosphamide and fludarabine with the addition of TBI supports *in vivo* expansion of adoptively transferred cytotoxic T-cells and NK cells (102–105). Also, T-cell depletion in the haploidentical HSCT setting is associated with more rapid NK cell immune reconstitution and strong NK-cell alloreactivity (78, 80, 83). The use of double (instead of single) unit CBT introduces another level of complexity due to lack of our understanding of three way KIR-L interactions among the recipient and the two CB grafts. In such cases, NK cells from the “dominant” CB unit are presumed to be contributing to the beneficial NK alloreactivity (94), but the role of NK cells in the non-dominant CB unit and their influence in mediating donor vs. recipient and graft vs. graft alloreactivity is not clear.

## Future Considerations

### Role of CMV in Influencing NK Cell Alloreactivity Post Transplantation

It is now well established that CMV infection or reactivation can reduce the risk of relapse after HSCT by enhancing NK cell maturation with increased CD56^dim^ population, shaping NK cells toward an activated phenotype with upregulation of NKG2C (activation) and KIR receptors and downregulation of NKG2A (inhibitory) along with increased expression of CD57, and creating “memory NK cells” (106–112). It is plausible that some of the discrepancies noted in the four CBT studies described above could be due to differential CMV reactivation among the different groups. As an example, significantly more patients in the KIR-L matched group were CMV seropositive compared with the KIR-L mismatched group in the Minnesota study (94). By contrast, in the Willemze study (97), equal numbers of patients were CMV seropositive in both KIR-L matched and mismatched groups, whereas the Japanese study (96) did not report on the CMV serostatus of their cohort.

### Role of Activating NK Cell Receptors and NK Haplotypes

Likewise, no study evaluated the role of activating KIRs on CBT outcomes, which are known to influence outcomes after other types of transplant (88, 113–117). The KIR genes are encoded as haplotypes, and individuals can be categorized as either haplotype A or B. Haplotype A individuals have more inhibitory KIRs, whereas haplotype B individuals carry more activating KIRs. The favorable effect of receiving grafts from haplotype B donors was demonstrated by Cooley et al. (117) in AML patients who underwent unrelated donor PB or BM HSCT. Patients whose donors had KIR-B/x haplotype (i.e., either homozygous or heterozygous B-haplotype) had improved relapse-free survival compared to those who received grafts from homozygous haplotype A donors. The impact of KIR haplotype on CBT outcomes has not been evaluated to date.

### Role of NK Cell Licensing Post Transplantation

Evaluation of the impact of NK cell licensing, a process by which NK cells gain functional competence (118), should be considered in future studies of CBT. The new MHC environment of the recipient can alter NK cell responsiveness (119). In an elegant study performed by Joncker et al. (119), adoptive transfer of mature functional NK cells from MHC-I wild-type mice into MHC-I-deficient mice resulted in loss of NK cell responsiveness. By contrast, transfer of hyporesponsive NK cells from MHC-I-deficient mice into MHC-I wild-type mice resulted in gain of functional competency. A recent study of unrelated donor PB/BM HSCT recipients provided clinical evidence for the significance of this dynamic process of licensing in humans. The authors found that patients who lacked cognate ligands involved in NK cell licensing for the inhibitory KIRs in the donor had significantly worse DFS, OS, and time to progression, compared with patients who had the ligands. This supports the principle that activated licensed NK cells are significantly more cytotoxic than the unlicensed cells and are more likely to mediate a strong graft vs. leukemia response (118).

## Cord Blood NK Cells in Cellular Therapies

In addition to the role of NK cells in CBT, the use of *ex vivo* purified and activated CB NK cells is also being explored for adoptive immunotherapy. Although numerous studies have been conducted using autologous or allogeneic (PB or BM) NK cells infusions [reviewed in Ref. (120–122)], no clinical study to date has reported on the use CB NK cells. A handful of clinical trials are currently underway to evaluate the feasibility, safety, and efficacy of CB NK cell adoptive immunotherapy in transplant and non-transplant settings (Table 2).

**Table 2:** **Ongoing clinical trials assessing the role of cord blood natural killer cells in adoptive immunotherapy**.

Clinical trial identifier	Trial phase	Disease	Type of transplant	Conditioning regimen	CB NK infusion day	Dose	Expansion
NCT01619761	I	AML, ALL, CML, MDS, NHL, SLL, CLL, NHL, HL, MM	DCBT	Fludarabine, melphalan, lenalidomide ± rituximab	Day 2	5 × 10^6^/kg	*Ex vivo* from 20% CB unit fraction
NCT01729091	I/II	Multiple myeloma	Autologous	Melphalan, lenalidomide (day 8–2)	Day 5	5 × 10^6^–1 × 10^8^/kg	*Ex vivo* expanded from thawed CB unit
NCT02280525	I	CLL refractory to at least 2 lines of standard chemoimmunotherapy, relapsed or refractory ALL, AML, CML, NHL, HL	Non-transplant setting	Fludarabine, cyclophosphamide, rituximab, and lenalidomide (day 5 to +14)	Day 0	Escalating doses (10^6^–10^8^/kg)	*Ex vivo* expanded from thawed CB unit
NCT01823198	I/II	Myeloid malignancies	Allogeneic	Busulfan, fludarabine	Day 8^a^	10^6^, 10^7^, 3 × 10^7^, or 10^8^/kg based on number of NK cells	*Ex vivo* + *in vivo* expansion with IL-2 (day 8–4)

*^a^NK cell source could be from matched related donor, haploidentical donor, or CB*.

*ALL, acute lymphoblastic leukemia; AML, acute myeloid leukemia; CB, cord blood; CLL, chronic lymphoblastic leukemia; CML, chronic myeloid leukemia; DCBT; double unit cord blood transplantation; HL, Hodgkin lymphoma; MDS, myelodysplastic syndromes; MM, multiple myeloma; NHL, non-Hodgkin lymphoma; NK, natural killer; SLL, small lymphocytic lymphoma*.

## Conclusion

Natural killer cells have a remarkable potential to kill cancer as well as virally infected cells. They are the first cells to reconstitute after HSCT (8–14), they facilitate engraftment (78), they do not cause GVHD, and they may even prevent this complication by eliminating host antigen-presenting cells and donor alloreactive T cells (83, 123). CB provides several distinctive benefits, and it is increasingly used as a source of CBT and cellular therapies. Resting CB NK cells are immature and are poorly cytotoxic compared with PB NK cells; however, these limitations can be overcome by *ex vivo* expansion using cytokines and feeder cells (48, 49, 52, 56, 124). A number of clinical studies are evaluating the feasibility, safety, and anti-tumor efficacy of adoptive immunotherapy with CB NK cells. The biological mechanism and tempo of NK cell alloreactivity after CBT, especially with double unit CBT has not been fully elucidated. Before the immunological reactivity of NK cells and KIRs could be targeted and exploited to improve the response to CBT, we will need to have a better understanding of the biological mechanisms involved in NK-mediated anti-leukemia response.

## Author Contributions

RM, ES, and KR wrote and edited the manuscript.

## Conflict of Interest Statement

The authors declare that the research was conducted in the absence of any commercial or financial relationships that could be construed as a potential conflict of interest.
